# Coexistence of Spontaneous Pneumomediastinum and a Parapharyngeal Space Tumor: A Case Report of a Patient Initially Referred for Descending Necrotizing Mediastinitis

**DOI:** 10.7759/cureus.69582

**Published:** 2024-09-17

**Authors:** Mariko Koizumi, Chiaki Takagi, Ryo Kawaura, Toshimitsu Ohashi, Masami Ohnishi

**Affiliations:** 1 Otorhinolaryngology-Head and Neck Surgery, Gifu Prefectural General Medical Center, Gifu, JPN; 2 Head and Neck Surgery-Otolaryngology, Ogaki Municipal Hospital, Ogaki, JPN; 3 Otolaryngology-Head and Neck Surgery, Gifu University Graduate School of Medicine, Gifu, JPN

**Keywords:** cervical abscess, descending necrotizing mediastinitis, parapharyngeal space tumor, pneumomediastinum (pm), spontaneous pneumomediastinum (spm)

## Abstract

Spontaneous pneumomediastinum is conventionally defined as a disease that does not result from trauma or underlying disease. In recent years, many patients have had some kind of triggering factor, such as sports or a strong cough. Herein, we report a case of mediastinal emphysema with a parapharyngeal tumor at the time of initial examination. Although the patient was referred to our hospital on suspicion of cervical abscess such as descending necrotizing mediastinitis by symptoms, it seemed that the cause was a spontaneous pneumomediastinum associated with a parapharyngeal tumor coincidentally. The pneumomediastinum resolved day by day, and the parapharyngeal tumor was extracted without recurrence, which turned out to be a pleomorphic adenoma. As far as we are concerned, there have been no reports of spontaneous pneumomediastinum associated with a parapharyngeal space tumor.

## Introduction

"Pneumomediastinum" is the term that defines the presence of air in the mediastinum. According to Hamman et al., spontaneous pneumomediastinum was considered without trauma or disease [1]; the definition has become more extensive due to recently reported cases. In this report, we experienced a case of spontaneous pneumomediastinum with parapharyngeal space tumor at the time of initial diagnosis. As far as we are concerned, there have been no reports of a parapharyngeal space tumor associated with spontaneous pneumomediastinum. When spontaneous pneumomediastinum is associated with a cervical tumor or abscess, descending necrotizing mediastinitis sometimes needs to be ruled out. We report the result of the case with a discussion of the literature.

## Case presentation

A 29-year-old male with no medical history at birth had an issue with the pharynx for a week and presented with a fever and sore throat. He had a fever of 38°C, which revealed swelling of the right soft palate. At that time, a plain computed tomography (CT) scan revealed a mass in the pharyngeal space and emphysema from the neck to the mediastinal. Suspected of a serious condition like pneumomediastinum with cervical abscess, the patient was referred to the emergency department of our hospital on the same day for further examination and advanced medical care.

Physical examination findings

The patient's height and weight were 171 cm and 50 kg, indicating a body mass index (BMI) of 17.0 kg/m^2^. During the oral examination, remarkable swelling of the right soft palate was seen, although no tonsillar hypertrophy or white adhesion of pus was observed. He complained of mild tenderness in the cricoid cartilage, although there was no evidence of hoarseness, gagging, dysphagia, labored breathing, wheezing, or stenotic sounds in the upper airway.

Diagnostic studies

Results of routine laboratory blood tests were almost normal; white blood cell count was 4000/µL (with neutrophils accounting for 55.7% and lymphocytes for 34.4%), lactate dehydrogenase was 194 IU/L, C-reactive protein (CRP) was 0.04 mg/dL, and glucose was 94 mg/dL. 

**Figure 1:**
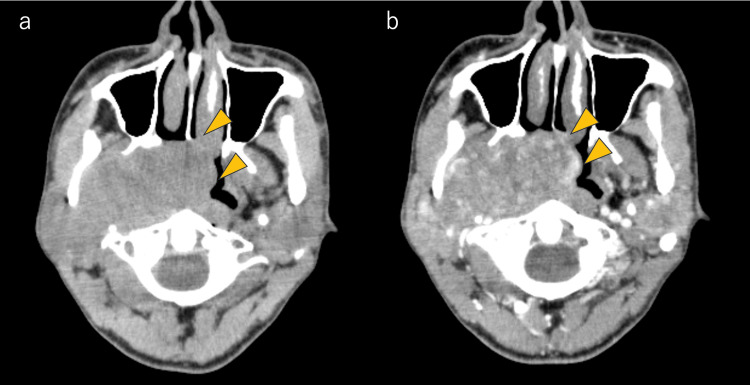
Images of computed tomography performed at the first visit Compared with a plain CT (a), the contrast-enhanced CT (b) revealed a lesion of 65 mm in diameter, indicating internal contrast heterogeneously (arrowhead).

**Figure 2:**
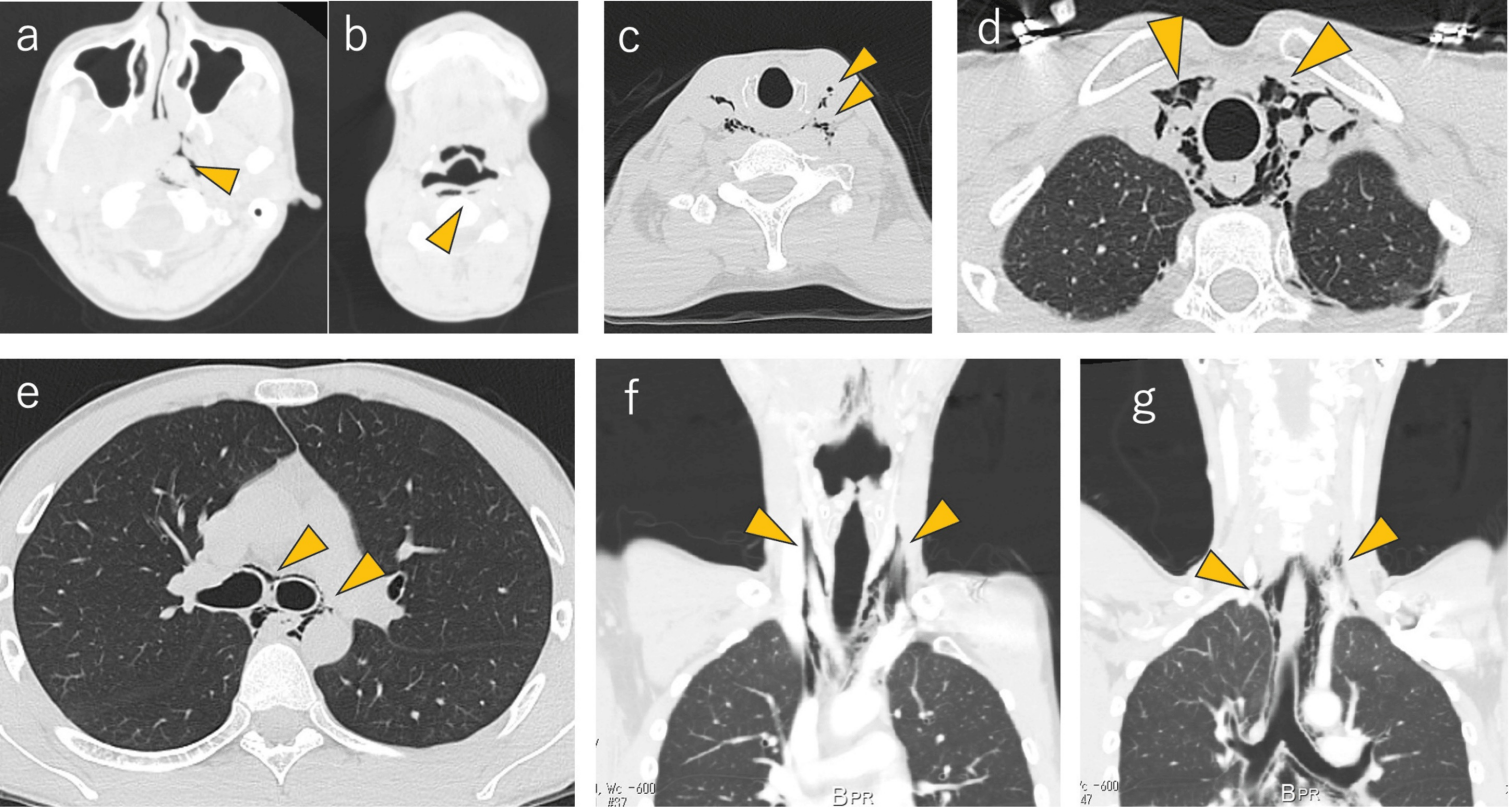
Images of emphysema on computed tomography A plain CT revealed the presence of emphysema (arrowheads) from the nasopharynx to the left parapharyngeal space (a), retropharyngeal space (b), both sides of carotid artery space (c.d), and mediastinum (e). Images (f) and (g) show the coronal view of the emphysema.

A contrast-enhanced CT of the cervical region revealed a lesion of 50x30x65mm in diameter, indicating internal contrast heterogeneously from the right side of the nasopharynx to the right lateral area of the hypopharynx (Figure 1). Also, a plain CT of the chest revealed emphysema distributed from the nasopharynx to the left parapharyngeal space, left and right carotid artery space, and mediastinum the height of the right atrium (Figure 2).

**Figure 3:**
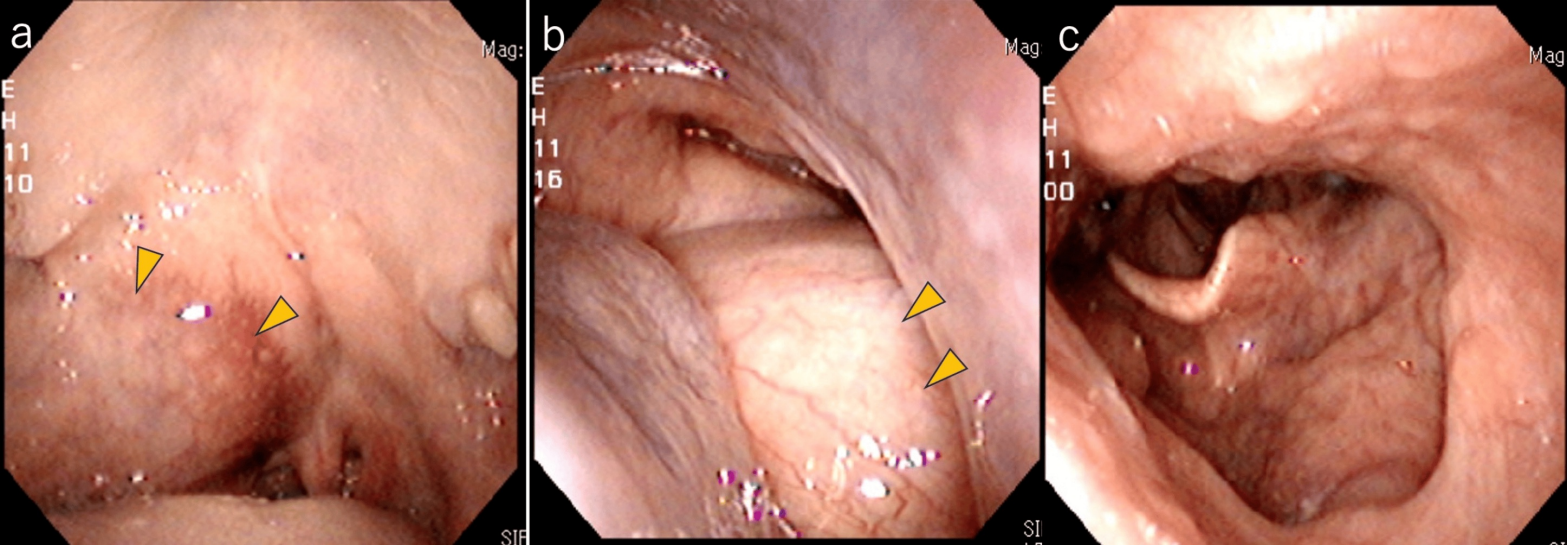
Images of laryngeal endoscopy Laryngeal endoscopy indicated the swelling (arrowheads) of the right soft palate (a) and blocked posterior nostrils (b); laryngeal edema was not seen (c).

Laryngeal endoscopy indicated the swelling of the right soft palate and a superficial mass lesion blocking the right posterior nostril. The right tympanic membrane was normal with no findings of the otitis media with effusion. Still, no laryngeal edema or swelling of the lateral wall of the pharynx was observed (Figure 3). Considering the presence of an abscess, a test puncture of the pharynx was performed; no pus was shown.

Based on these findings, it was not assumed that his disease was peritonsillar or cervical abscess. The pneumomediastinum was considered to be spontaneous because there was no underlying disease such as asthma or other causes such as traffic trauma or esophageal rupture.

**Figure 4:**
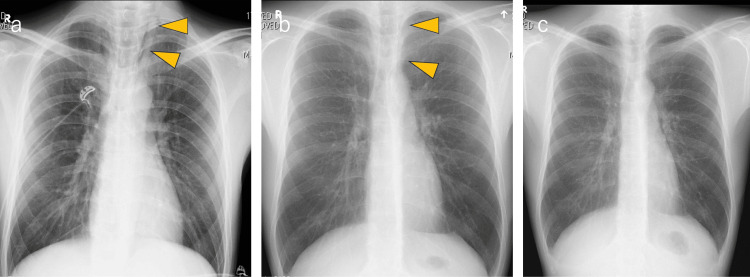
Pneumomediastinum on chest X-rays The spontaneous pneumomediastinum (arrowheads) gradually disappeared from day one (a) to day two (b) and completely disappeared by day nine (c).

He was admitted to our department on the same day and treated with antibiotics (ampicillin-sulbactam, intravenous, 3.0 g, three times per day) under hospitalization after consultation with the respiratory physician. Two days later, his subjective symptoms and mediastinal emphysema on the chest X-ray gradually improved. The spontaneous pneumomediastinum completely disappeared on day nine (Figure 4), hence, follow-up by the respiratory physician was finished. 

**Figure 5:**
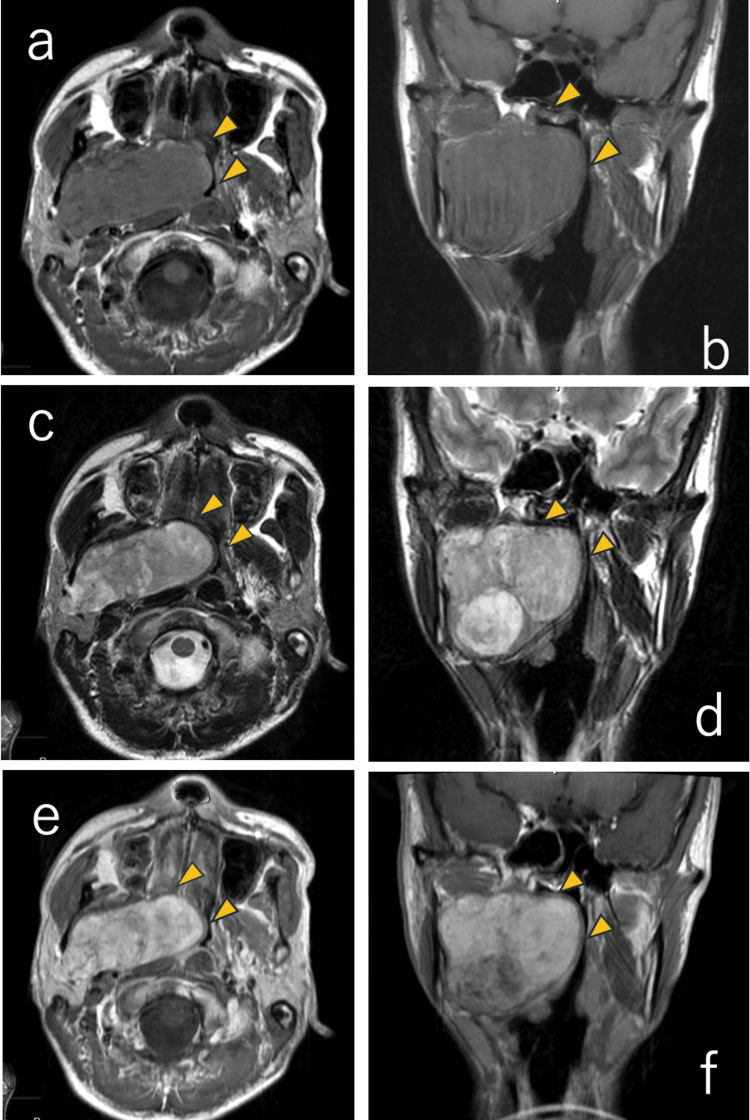
Magnetic resonance imaging reports The MRI indicated an 80-mm-long neoplastic lesion (arrowheads) occupying the oral cavity to the parapharyngeal space. (a) and (b): T1-weighted image; (c) and (d): T2-weighted image; (e) and (f): Gadolinium-enhanced T1 weighted images show mild enhancement

**Figure 6:**
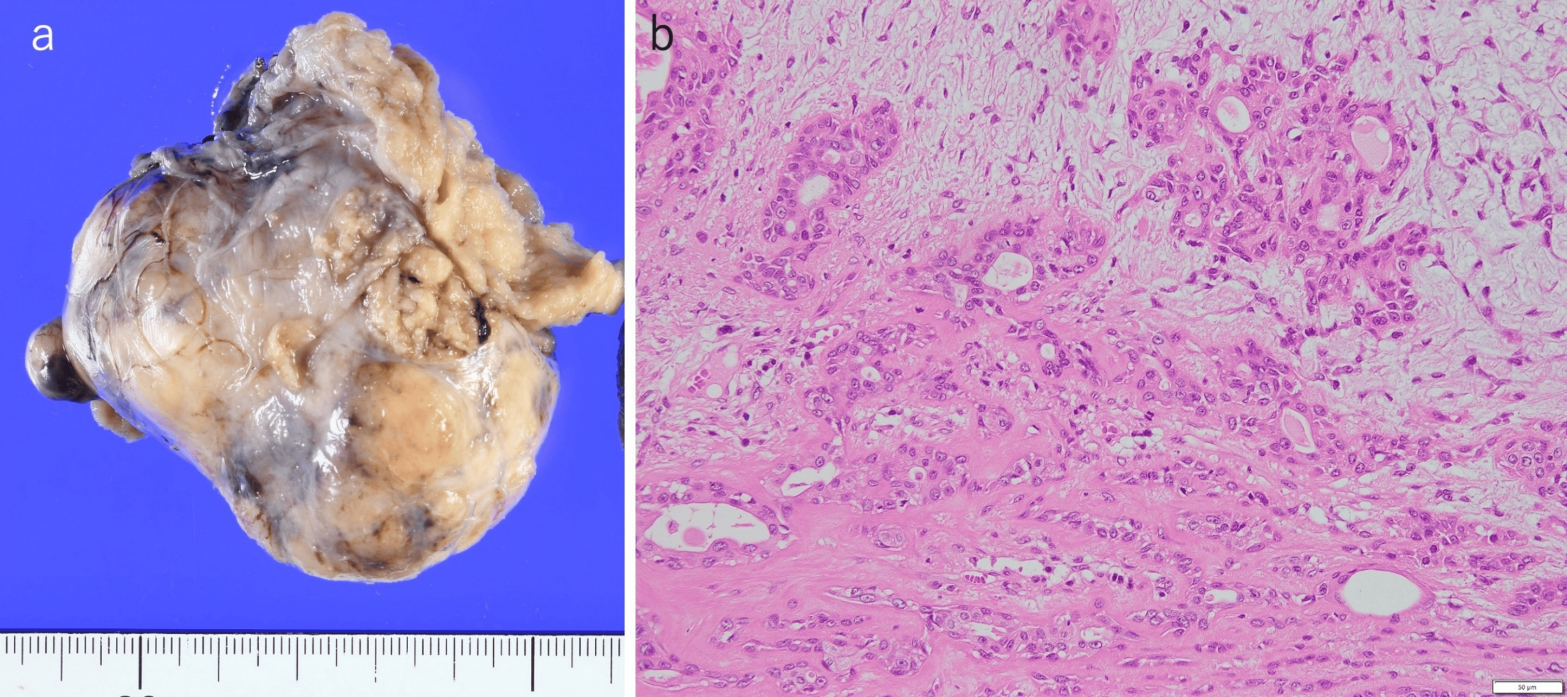
Pathological images of tumor The tumor (a) was surrounded by a capsule with cartilage-like interstitium, without cellular atypia (b). The scale bar indicates 50 um.

For the pharyngeal lesion, magnetic resonance imaging (MRI) was performed, indicating an 80-mm-long neoplastic lesion occupying the oral cavity to the parapharyngeal space (Figure 5). As the tumor was compressing the muscles of the styloid process posteriorly, it was considered to be a pre-styloid tumor of the parapharyngeal space. We performed fine needle aspiration cytology (FNAC) with short spindle-shaped myoepithelial cells and mucous-like stroma, suggesting a high possibility of pleomorphic adenoma. Two months after the first admission, he underwent a resection of a parapharyngeal space tumor under general anesthesia. The surgery was performed by the trans-orbital method. After the elevation of the skin valve, the tumor extending from the deep lobe of the parotid gland to the parapharyngeal space was dissected. The tumor was removed en bloc by compression without damaging the pharyngeal mucosa. The operation time was one hour and 57 minutes. The pathological diagnosis of the excised specimen (Figure 6) was a pleomorphic adenoma, the same as the preoperative diagnosis. Postoperatively, mild paralysis of the mandibular border branch of the facial nerve was observed, and first bite syndrome was temporarily treated with medication, which disappeared at two months postoperatively. Both the tumor and spontaneous pneumomediastinum have not recurred.

## Discussion

Spontaneous pneumomediastinum is defined as a disease of sudden onset in a healthy person without any underlying disease due to some non-morbid triggers [2]. It is a relatively rare condition, occurring in 0.003% to 0.049% of hospitalized patients and 0.0022% of emergency department patients [3]. Macklin's theory [4] has long been recognized as the mechanism of the onset of the disease: alveoli rupture due to increased intra-alveolar pressure and leaked air moves to the pulmonary portal, mediastinum, and subcutaneous area along the vascular capsule of the pulmonary vascular sheath. Small blebs, where the alveoli rupture may occur, are present even in normal lungs [5]. Also, there is another hypothesis that indicates pneumomediastinum is caused by the pressure gradient between the intrapleural space and mediastinum. Increased intrapleural pressure causes air leaks, resulting in positive pressure pneumomediastinum. On the contrary, the reduction of mediastinal fat caused by body weight loss reduces the mediastinal pressure and causes negative pressure pneumomediastinum [6]. According to a report on the clinical features of spontaneous pneumomediastinum [7], the mean age of onset was 20.8 years old, the average BMI was 19.5 kg/m^2^, and the male-to-female ratio was 52:23, indicating that the disease was more common in young men of thin build and tall stature, just like in this case. Initial symptoms of spontaneous pneumomediastinum were snow grasping sense (62%), chest pain (60%), sore throat (36%), and neck pain (30%). Conservative therapy is widely chosen for the treatment, including rest and administration of antibiotics to prevent mediastinitis. Recently, however, there has been a review of the use of antibiotics only in cases of elevated amounts of white blood cells or levels of CRP [8]. Meanwhile, surgical procedures such as drainage and tracheotomy are often performed in cases of mediastinitis or complications of secondary pneumothorax. Although the prognosis of pneumomediastinum is generally good with a recurrence rate of 2.5% to 5.4% [3], there have been reports of tension pneumothorax [9] and cases of respiratory arrest due to subcutaneous emphysema [10], which is the reason why hospitalization is necessary.

Parapharyngeal space tumors account for 0.5% of all head and neck tumors [11]. The parapharyngeal space is an inverted cone-shaped space located from the base of the skull to the level of the hyoid bone. It is surrounded anteriorly by the medial pterygoid muscle, laterally by the parotid deep lobe capsule, and medially by the pharyngeal constrictor muscle; important structures, such as the vagus nerve, accessory nerve, sublingual nerve, sympathetic nerve, and internal jugular vein, run through it [12]. Parapharyngeal space tumors are classified into the anterior and posterior stem-penetrating regions based on their positional relationship to the styloid process. Most tumors originating from the pre-styloid region are pleomorphic adenomas (93.8%), while tumors from the posterior region are schwannomas (87.5%); all malignant tumors are considered to be derived from the pre-styloid region [11]. For this reason, surgery should be aggressively considered for tumors originating from the pre-styloid region, while careful evaluation is well required for tumors originating from the post-styloid region [12]. In this case, the location of the tumor and histopathology were typical for parapharyngeal space tumors originating from the anterior styloid process region, so we had a choice of surgery as the treatment.

As far as we are concerned, there were no reports of spontaneous pneumomediastinum associated with a parapharyngeal space tumor (based on a PubMed search for "parapharyngeal space tumor (spontaneous) pneumomediastinum"). In terms of the field of otorhinolaryngology, several reports of spontaneous pneumomediastinum as surgical complications are known, for example, subcutaneous emphysema and spontaneous pneumomediastinum after tonsillectomy under local anesthesia [13]. Araki et al. reported that cervical subcutaneous emphysema, as a complication, occurs in 1.1% to 3.6% of patients with transoral video laryngoscopic surgery (TOVS), which has recently become common in the field of head and neck cancer [14, 15]. Although no cases of spontaneous pneumomediastinum after TOVS are reported, we should pay attention to them in the postoperative period in addition to subcutaneous emphysema.

In general, both spontaneous pneumomediastinum and parapharyngeal space tumors are not well known except for otolaryngologists. In this case, non-otolaryngologists treated the patient at the time of initial examination at both the previous hospital and our hospital; the first problem was to determine whether it was not descending necrotizing mediastinitis or spontaneous pneumomediastinum coexisting with a parapharyngeal space tumor. According to Estrera et al. [16], the definition of descending mediastinitis is (1) the presence of severe infection, (2) typical imaging findings, (3) infection of the mediastinum with necrosis confirmed at surgery or autopsy, and (4) the presence of oropharyngeal infection and necrotizing mediastinitis. Findings of infection in the oropharyngeal region are often difficult for non-otolaryngologists and residents, who are required to evaluate blood tests and imaging studies appropriately.

In this case, whether the tumor is related to the development of spontaneous pneumomediastinum is the point of discussion. If that is true, the distribution of emphysema will be mainly around the tumor, or there will be a clear mechanism that shows the relationship between the two diseases. Also, the epidemiological characteristics are consistent with the favorable nature of spontaneous pneumomediastinum. However, at present, it is impossible to confirm the relationship between the two diseases, and we can only conclude that it was accidental.

Also, fortunately, as inflammatory findings on blood tests were mild and there were no remarkable findings except for swelling due to the tumor in the pharynx, it was easy to diagnose for otolaryngologists. Nevertheless, it is still important to remain calm and accurately evaluate laboratory findings even when encountering atypical findings in an environment that differs from the usual outpatient setting, such as an emergency department.

## Conclusions

We report a case of spontaneous pneumomediastinum and a parapharyngeal space tumor, which was initially suspected to be descending necrotizing mediastinitis, but it is concluded that this is the case of spontaneous pneumomediastinum occurring coincidentally with a tumor. Both diseases occur infrequently, and non-otolaryngologists may have difficulty in diagnosis. The relationship between the two diseases is still unclear and needs to be further investigated.
